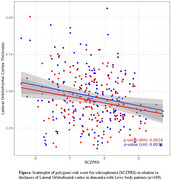# Genetic risk for psychiatric disorders and prefrontal lobe thickness in a memory clinic population

**DOI:** 10.1002/alz70856_105421

**Published:** 2026-01-07

**Authors:** Jenna Najar, Ellen Dicks, Afina W. Lemstra, Wiesje M. van der Flier, Betty M. Tijms, Yolande A.L. Pijnenburg, Sven J van der Lee, Lianne M. Reus

**Affiliations:** ^1^ Genomics of Neurodegenerative Diseases and Aging, Human Genetics, Amsterdam UMC, Amsterdam, Netherlands; ^2^ Psychiatry and Neurochemistry, Sahlgrenska Academy, University of Gothenburg, Gothenburg, Sweden; ^3^ Alzheimer Center Amsterdam, Neurology, Vrije Universiteit Amsterdam, Amsterdam UMC location VUmc, Amsterdam, Netherlands; ^4^ Amsterdam Neuroscience, Neurodegeneration, Amsterdam, Noord‐Holland, Netherlands; ^5^ Alzheimer Center Amsterdam, Neurology, Amsterdam UMC Location VUmc, Vrije Universiteit Amsterdam, Amsterdam, Netherlands; ^6^ Alzheimer Center Amsterdam, Neurology, Vrije Universiteit Amsterdam, Amsterdam UMC location VUmc, Amsterdam, Noord‐Holland, Netherlands; ^7^ Alzheimer Center Amsterdam, Department of Neurology, Amsterdam UMC, location VUmc, Amsterdam, Netherlands; ^8^ Center for Neurobehavioral Genetics, Semel Institute for Neuroscience and Human Behavior, David Geffen School of Medicine, University of California, Los Angeles, Los Angeles, CA, USA, Los Angeles, CA, USA

## Abstract

**Background:**

Psychiatric disorders and dementia share overlapping clinical and genetic factors (e.g., *SNCA, CLU, and APOE*), and both conditions implicating the prefrontal cortex (PFC). We examined the association between genetic liability for psychiatric disorders and PFC thickness in Alzheimer's disease (AD), dementia with Lewy bodies (DLB), frontotemporal dementia (FTD), and cognitively healthy controls.

**Method:**

We analyzed 2,538 individuals (1,258 AD, 169 DLB, 238 FTD, and 873 controls) from the Amsterdam Dementia Cohort. 3D T1w images were processed with FreeSurfer (version 7.1.1) and labels for PFC correspond to those from the Desikan‐Kiliany atlas. The segmentation of all images were visually quality checked. Polygenic risk scores (PRS) for depression (MDDPRS), bipolar disorder (BDPRS), schizophrenia (SCZPRS), and autism (ASDPRS) were calculated using LDpred2, with weights from independent GWAS. Linear mixed models were used to test associations between PRS and PFC thickness, using PRS*hemisphere, PRS*diagnostic group, age, sex, scanner model, and total gray matter volume as fixed effects and hemisphere as random effect.

**Result:**

No significant main effects were observed between PRS and PFC thickness. Hemisphere did not influence the relationship for BDPRS, SCZPRS, or ASDPRS. MDDPRS interacted with hemisphere in relation to PFC, showing positive associations in the left hemisphere and negative in right, although no association reached significance. SCZPRS interacted with DLB case‐control status in relation to lateral orbitofrontal cortex (OFC) thickness, showing that higher SCZPRS associated with reduced lateral OFC thickness in DLB patients (B=‐0.05, *p* = 0.003), but not in controls (B=0.003, *p* = 0.6). Additionally, ASDPRS interacted with FTD in relation to PFC regions, although stratified models did not reach significance.

**Conclusion:**

The finding that SCZPRS is associated with reduced lateral OFC thickness in DLB patients suggests a potential genetic basis for psychotic symptoms in DLB and contributes to understanding the neurobiological overlap between DLB and schizophrenia.